# Carbon Emissions in the SAARC Countries with Causal Effects of FDI, Economic Growth and Other Economic Factors: Evidence from Dynamic Simultaneous Equation Models

**DOI:** 10.3390/ijerph18094605

**Published:** 2021-04-27

**Authors:** Rashid Latief, Yusheng Kong, Sohail Ahmad Javeed, Usman Sattar

**Affiliations:** 1 School of Finance and Economics, Jiangsu University, Zhenjiang 212013, China; 2 College of Finance, Nanjing Agricultural University, Nanjing 210095, China; 2017218009@njau.edu.cn; 3 College of Law and Political Science, Zhejiang Normal University, Jinhua 321004, China; usman@zjnu.edu.cn

**Keywords:** FDI, carbon emissions (CE), economic growth (EG), SAARC countries

## Abstract

South Asian Association for Regional Cooperation (SAARC) countries like other developing countries are the major destination for foreign investors. At the same time, these countries are facing different climate change challenges. This study aims to inspect the economic determinants of carbon emissions (CE) and dynamic causal interaction of CE with foreign direct investment (FDI), economic growth (EG), and other economic factors using panel cointegration test, dynamic ordinary least squares (DOLS) and vector error correction model (VECM) for the SAARC countries. To make the homogenous analysis, we examined the association among variables for the individual country and as a group for the period 1990 to 2016. The panel results of this study confirmed the presence of the unidirectional causal association of EG with CE. The panel results of other economic factors confirmed the causality of urban population (UP) and energy consumption (EC) with CE. Moreover, the panel results of domestic capital (DS) and inflation rate (INF) confirmed the causal association with EG. Finally, the panel results of DS revealed a causality with FDI. Based on the above results, some policy guidelines are proposed.

## 1. Introduction

Climate change is an area-sensitive issue. The spatial dimensions of climate change are increasingly becoming a matter of concern for researchers and policymakers as urban areas and developed countries are significant contributors to CE as compared to least developed countries [1]. Therefore, climate change adaptation requires a global level joint effort. In 2015, an international conference on climate change was held in France, which was attended by the top global leaders from 196 countries including the United States [2]. They agreed to adopt nationally determined contributions (NDCs) to curb the climate change issue—Paris Agreement [3]. Many countries including the European Union and China have made jointly possible efforts to curb global climate changes [4]. However, the United States has announced its withdrawal from the Paris Agreement in June 2017 after President Trump took office [5]. The US withdrawal from the Paris Agreement, being the largest per capita carbon emitter country in the world, has great economic led environmental implications for all other countries [6].

Developing countries with huge land, population, and growing economies have great potential for carbon reduction but developing countries require huge financial and technical support to utilize their physical capital for a global cause as noted in the Paris accord. Most of the developing countries are facing socio-economic and political instability, which creates hurdles for capital accumulation and using the available resources for their global commitments. As a result, the developing countries move towards international aid, portfolio flows, and FDI to achieve EG. FDI has comparatively more advantages than other sources; it helps to achieve constant funds flow, increased capacity, new job opportunities, and better trade flow in the host countries [7].

The South Asian region consists of varied environmental zones and experiences different impacts of climate change. Carbon emission (CE) has consistently been increased in the South Asian region in recent years. India and Pakistan are the main contributors to the pollution of the environment of the region as they produce a high level of carbon dioxide (CO_2_) [8]. There are several economic contributing factors to CE such as foreign investment, economic growth (EG), energy consumption (EC), labor force (LF), urban population (UP), inflation (INF), tourism, transport, etc., [9,10,11,12]. The environmental changes in this region are occurred in the form of global warming, increasing the seawater levels, variations in the pattern of rainfalls, and rising the frequency of cyclones or floods [13]. The geophysical and demographic changes in this region are more vulnerable to environmental changes. The effects of environmental changes are evident in agriculture, forests, water resources, and ecosystems. These changes may create a major threat to the safe living of billions of people living in this region [14].

SAARC countries like other developing countries have also been attracting foreign investment in the last few decades. They implemented economic policies and reforms to decrease hurdles in the way of foreign investment. However, they were unsuccessful in their motive to attract a higher level of foreign investment as compared to other countries in the East/Southeast Asian regions due to different factors. Later, the policymakers of the South Asian region realized that comprehensive policies are needed for upgrading the technology, human capital, scale of production, and integrated production system. SAARC countries have the potential to increase foreign investment by highlighting the positive aspects such as single-digit inflation, high EG, skilled and cheap workforce, and many others [15].

Here, a basic question is raised as to who should be accountable for global warming, either the countries that are involved in the production activities or the countries where these goods are consumed? The developing countries may suffer from a polluted environment caused by carbon emission (CE). This is a matter of concern, which needs extensive debate. Presently, the research on the nexus of the environment with economic openness has emphasized the pollution haven hypothesis (PHH), which expects that free trade between countries may cause significant pollution to the developing countries as foreign firms move towards the developing countries after facing strict environmental regulations in their home countries [16].

Indeed, FDI considerably increases the economic development of particularly those countries, which do not have capital, advanced technology, and skills to utilize their natural resources for fulfilling their need [17]. The positive externalities of FDI are significant for the hosting countries; while, multinational firms set goals to achieve higher profit and growth [18]. Although FDI helps to raise the level of economic growth (EG), at the same time, it may also be a cause for concern by considering FDI-led growth from an environmental perspective. The relationship between environment and FDI could be positive or negative [17].

Most of the previous studies about the role of FDI from an environmental perspective have focused on developed countries as compared to developing countries, but the results are conflicting (See Bakhsh, Rose, Ali, Ahmad and Shahbaz [12], Waqih, et al. [19], Sun, et al. [20], Chandran and Tang [21], Behera and Dash [22], Pao and Tsai [23], and Balsalobre-Lorente et al. [24]). This study contributes to the present literature in the following ways: (i) It analyzes the causal association between FDI, EG, and CE in terms of environmental Kuznets curve (EKC) for SAARC countries. (ii) It considers the role of important economic determinants of CE, selected from developing economies such as financial development (FD), urban population (UP), energy consumption (EC), domestic capital (DS), labor force (LF), inflation (INF), the exchange rate (EX) and trade openness (TD). (iii) It uses the possible most updated sample data based on the availability. (iv) It investigates the causal association among variables for the group and individual countries. (v) It applies the panel dynamic ordinary least squares (DOLS) to find out important economic features and long-run association for the selected panel of countries. (vi) The empirical findings of this study will be constructive for policymakers to obtain a better understanding of FDI, CE, and EG nexus to design the policies to deal with environmental and economic challenges.

The rest of the paper is organized as follows: the second section includes a review of previous work from relevant theoretical and empirical perspectives. The third section describes the sample, variables, and analysis techniques used in this study. The fourth section provides the results and discussion on the association between FDI, EG, and CE. The fifth section concludes the study with policy implications.

## 2. Theoretical Framework and Literature Review

### 2.1. Foreign Direct Investment (FDI) and Carbon Emissions (CE)

Theoretically, the connection between FDI and the environment is based on three main hypotheses: pollution-haven hypothesis, pollution-halo hypothesis, and scale effect hypothesis. According to the *pollution-haven hypothesis*, countries with weak environmental regulations can attract more foreign firms for investment. Foreign firms with profit-oriented purposes may avoid following the costly environmental regulations of their home countries. Therefore, this hypothesis suggests that FDI may lead to the risk of further polluting the environment [19,23]. According to the *pollution-halo hypothesis*, by following the universal environmental standards, foreign firms tend to transfer the environment-friendly technology to the host country through FDI in the host country. In this scenario, the effect of FDI may be negative or positive, which could lead to improvement or further deterioration of the environment [23,24]. The third perspective used to investigate the association between FDI and the environment is the *scale-effect hypothesis*. According to this hypothesis, foreign firms operate with full capacity in the host country, and FDI significantly contributes to the host economy, consequently, the environmental quality is significantly reduced [23].

A substantial amount of empirical evidence exists in the literature to determine the nexus between FDI and CE. Studies that are concerned with a single country to the association of CE and FDI contain Bakhsh, Rose, Ali, Ahmad and Shahbaz [12], Wang, et al. [25], Salahuddin, et al. [26], and Hajilary, et al. [27]. Studies that are concerned with multiple countries to the nexus of carbon emissions (CE) and FDI include Abdouli and Hammami [28], Baek [29], Chandran and Tang [21], Behera and Dash [22], Doytch and Uctum [30], Rafindadi, et al. [31], Hakimi and Hamdi [32], Hanif, et al. [33], Lee [34], Omri, et al. [35], Shahbaz, et al. [36], Kivyiro and Arminen [37], Pao and Tsai [23], Kahouli and Omri [38], Waqih, Bhutto, Ghumro, Kumar and Salam [19], and Sun, Tariq, Haris and Mohsin [20].

The studies that considered a single country to study the connection of carbon emissions (CE) with FDI, for instance, Bakhsh et al. [12] revealed a positive relationship of FDI and EG with carbon emissions (CE) with the help of Pakistani data for the period 1980 to 2014. They employed a simultaneous equation model to analyze the association between FDI, EG, and carbon emissions (CE). Moreover, Wang et al. [25] empirically analyzed the interactions between socioeconomic variables with the help of Chinese data for the period 1980 to 2015. Using VECM and impulse response function, they revealed the significant effect of investment on CE in the long run.

Salahuddin et al. [26] empirically analyzed the effect of FDI, energy consumption (EC), and financial development (FD) on carbon emissions (CE) with the help of Kuwaiti data for the period 1980 to 2013. Using the autoregressive-distributed lag (ARDL) technique, they revealed that FDI and other variables increase carbon emissions (CE). The findings of VECM revealed that FDI and other variables strongly Granger cause carbon emissions (CE). Furthermore, Hajilary et al. [27] revealed the significant association between FDI and carbon emissions (CE) with the help of data from Iran for the period 1976 to 2016. They used the partial least square (PLS) model to observe the relationship between variables.

The studies that considered multiple countries to examine the connection of carbon emissions (CE) and FDI, for instance, Abdouli and Hammami [28] used the VAR model and panel data of 17 the Middle East and North Africa (MENA) countries for the period 1990 to 2012. They analyzed the causality between FDI, carbon emissions (CE), and EG. The results of the study showed the unidirectional causality from EG to CE, and supported the unidirectional connection of FDI with CE. Besides, Baek [29] estimated the effect of FDI and other variables on the environment by using the data of 5 ASEAN countries for the period 1981 to 2010. He employed a pooled mean group estimator of the panel dynamic model to explore the association between these variables. The results of the study supported the pollution haven hypothesis (PHH) by endorsing the positive association of FDI with CE.

In the same way, Chandran and Tang [21] investigated the environmental Kuznets curve (EKC) hypothesis by using data of five Association of Southeast Asian Nations (ASEAN) countries. They employed Granger causality tests to examine the influence of FDI and EC on CE. The results of the study demonstrated the bidirectional causal connection between FDI, carbon emissions (CE), and energy consumption (EC) in the case of Malaysia and Thailand. Furthermore, Behera and Dash [22] analyzed the association between carbon emissions (CE), FDI, urban population, and EC by using panel data of 17 Asian countries for the period 1980 to 2012. They employed dynamic OLS (DOLS) and fully modified OLS (FMOLS) to analyze the association between these variables. The results of the study demonstrated co-integration among variables in all groups of countries, irrespective of their national income level.

Additionally, Waqih et al. [19] examined the pollution haven hypothesis and environmental Kuznets curve by selecting the sample of SAARC countries. They revealed a significant association of FDI with CE. They used panel data from 1986 to 2014 and empirically analyzed it by using nascent techniques, panel ARDL, and FMOLS. Additionally, Sun et al. [20] found a long-run positive association of FDI with CE, and a negative short-run association of FDI with CE in the SAARC countries by employing the panel ARDL model.

### 2.2. Carbon Emissions (CE) and Economic Growth (EG)

Kuznets [39] portrayed the EKC hypothesis and suggested an inverted-U-shaped association between income inequality and income. In the same way, the same inverted U-shaped association was extracted to elaborate the association between environment and income (per capita) in the 1990s [40]. Generally, the association between EG and environment is elaborated with EKC, while EG is the main cause of increased environmental degradation, but after a certain point of time, their association follows an inverted U shape trend [41].

The inverted U-shaped association between environment and EG is based on three types of effects such as scale effect, composition effect, and technique effect [40]. According to the scale effect, EG negatively affects the environment. Economic growth (EG) is based on the production level in a country. By increasing the production level in the country, the environment of the country becomes more polluted. Contrary to the scale effect, the composition effect suggests the positive association of EG with the environment. At the early stage, the environment of the country is degraded during economic development as a result of changing the economic structure of the country from agriculture to heavy manufacturing sectors. At the later stage, environmental quality is decreased as the economic structure moves towards the services and light manufacturing sectors. Lastly, the technique effect implies that EG helps to decrease environmental quality as green technologies are adopted along with economic growth. According to EKC, the scale effects are dominated at the early stage by showing adverse effects of economic growth on the environment, but other compositions and technique effects are shown at the later stage [42].

A significant amount of literature has concentrated on the association between carbon emissions (CE) and economic growth (EG). The studies that concentrated on a single country to inspect the association between these variables contain Ghosh [43], Hajilary et al. [27], Lotfalipour, et al. [44], Ozturk and Acaravci [45], Jalil and Mahmud [46], and Salahuddin et al. [26]. The studies that focused on multiple countries to inspect the association between these variables contain Arouri, et al. [47], Hossain [48], Govindaraju and Tang [49], Sebri and Ben-Salha [50], Pao and Tsai [23], Abdouli and Hammami [28], Omri et al. [35], Pandey and Mishra [51], Anser, et al. [52], and Rehman and Rashid [53].

The studies that considered a single country to study the connection of carbon emissions (CE) and EG, for instance: Ghosh [43] revealed the bidirectional short-run causality between carbon emissions (CE) and EG by using ARDL and Johansen co-integration along with the Indian data from 1971 to 2006. Moreover, Hajilary et al. [27] revealed the positive association between non-oil GDP and CE, and they found an insignificant association of EG with carbon emissions (CE) by using data of Iran for the period 1976 to 2016. They used the partial least square (PLS) model to examine the association between variables.

Furthermore, Lotfalipour et al. [44] found the unidirectional causality between EG and CE in Iran by using data from 1967 to 2007. They employed the Toda-Yamamoto method to study the association between these variables. Moreover, Ozturk and Acaravci [45] revealed the connection between carbon emissions (CE), EG, and other variables, but they failed to find significant Granger causality between carbon emissions (CE) and EG for Turkey by using data for the period 1968 to 2005.

The studies that considered multiple countries to study the connection of carbon emissions (CE) and EG, for instance, Arouri et al. [47] found a quadratic relationship of carbon emissions (CE) with EG in the Middle East and North African countries, and also the presence of EKC hypothesis in most of the countries, although they found varied results in some countries by using the data for the period 1981 to 2005. Besides, Hossain [48] found the unidirectional relationship by using the data of 10 newly industrialized countries for the period from 1971 to 2007, and he used Johansen bi-variate co-integration model to examine the association between variables.

Additionally, Govindaraju and Tang [49] used a panel of China and India and employed causality tests. The findings revealed the bidirectional causal connection between carbon emissions (CE) and EG. Moreover, Sebri and Ben-Salha [50] found bidirectional causality between CE and EG by using the data of BRICS countries for the period 1971 to 2010, and they used ARDL and VECM to investigate the connection between variables.

Moreover, Pandey and Mishra [51] found the unidirectional causal association from EG to CE in the case of SAARC countries by using the panel VECM model. Additionally, Anser et al. [52] revealed a positive association between EG and CE in the SAARC countries by using an augmented STIRPAT model for the period 1994–2013. Likewise, Rehman and Rashid [53] observed the bidirectional causality between CE and EG by selecting the sample of SAARC countries. They used data for the period 1960 to 2015 and examined it by using fully modified OLS and dynamic OLS.

## 3. Data, Sample, and Research Methods

### 3.1. Sample and Data Sources

The sample of this study is comprised of five SAARC countries, namely Bangladesh, India, Nepal, Pakistan, and Sri Lanka to examine the interaction between FDI, EG, and carbon emissions (CE). We collected the annual data from World Development Indicators (WDI) [54], SAARC energy outlook 2030 [55], the international monetary fund (IMF) [56], and Bruegel Dataset [57] for the period 1990 to 2016 to investigate the connection between variables of interest in the group and individual countries.

### 3.2. Variables Measurement

This study used several variables including carbon emissions (CE) measured by carbon dioxide emissions (metric tons per capita), net FDI inflows (current USD), economic growth (EG) measured by GDP per capita (current USD), financial development (FD) measured by domestic credit to the private sector (% of GDP), urban population (UP) measured by urban population growth (annual %), energy consumption (EC) measured by per capita energy use (kg of oil equivalent), domestic capital (DS) measured by gross fixed capital formation (% of GDP), total labor force (LF), real foreign exchange rate (EX), trade openness (TD) measured by the total trade (% of GDP) and inflation rate (INF) measured by consumer price index in line with the prior studies conducted by Lin and Nelson [58], Omri et al. [35], Latief, et al. [59] and Shahbaz et al. [17].

### 3.3. Model Specification

To analyze the determinants of carbon emissions (CE) and interaction between CE, FDI, and EG, we adopted the function (1) in line with a prior study conducted by Shahbaz et al. [17], whereby carbon emissions (CE) is based on two key explanatory factors including FDI and EG and other important controlling factors such as financial development (FD), urban population (UP), and energy consumption (EC):

CO_2_ = f(GDPP, FDI, FD, UP, EC) (1)

where CO_2_, GDPP and FDI denote the carbon emissions (CE), economic growth (EG) and foreign direct investment (FDI) respectively. The above-mentioned function (1) can also be written in log-linear with time specification as follows:

(2) $$lnCO_{2,t}=\xi_{0}+\alpha_{1}lnGDPP_{t}+\alpha_{2}lnFDI_{t}+\alpha_{3}lnFD_{t}+\alpha_{4}lnUP_{t}+\alpha_{5}lnEC_{t}+\eta_{t}$$

Based on theoretical literature and prior empirical studies conducted by Lin and Nelson [58] and Omri et al. [35], we developed the following simultaneous equations for examining the interaction between carbon emissions (CE), FDI, and economic growth (EG). Since this study used panel data, the panel form of Equation (2) is given below:

(3) $$lnCO_{2,it}=\xi_{0}+\alpha_{1}lnGDPP_{it}+\alpha_{2}lnFDI_{it}+\alpha_{3}lnFD_{it}+\alpha_{4}lnUP_{it}+\alpha_{5}lnEC_{it}+\eta_{it}$$

(4) $$lnGDPP_{it}=\beta_{0}+\beta_{1}lnCO_{2,it}+\beta_{2}lnFDI_{it}+\beta_{3}lnDS_{it}+\beta_{4}lnLF_{it}+\beta_{5}lnINF_{it}+\mu_{it}$$

(5) $$\begin{array}{c} lnFDI_{it}=\gamma_{0}+\gamma_{1}lnGDPP_{it}+\gamma_{2}lnCO_{2,\;it}+\gamma_{3}lnDS_{it}+\gamma_{4}lnLF_{it}+\gamma_{5}lnEX_{it}\\ +\gamma_{6}lnTD_{it}+\pi_{it} \end{array}$$

From Equations (3)–(5), $lnCO_{2,it}$ represents the logarithm of carbon emissions, $lnGDPP_{it}$ represents the logarithm of economic growth, $lnFDI_{it}$ represents the logarithm of foreign direct investment, $lnFD_{it}$ represents the logarithm of financial development, $lnUP_{it}$ represents the logarithm of the urban population, $lnEC_{it}$ represents the logarithm of energy consumption, $lnDS_{it}$ shows the logarithm of domestic capital, $lnLF_{it}$ represents the logarithm of the labor force, $lnINF_{it}$ shows the logarithm of the inflation rate, $lnEX_{it}$ shows the logarithm of the real exchange rate, $lnTD_{it}$ represents the logarithm of trade openness. $\xi_{0}$,$\beta_{0}$, and $\gamma_{0}$ represent the heterogeneity among cross-sections, $\eta_{it}$, $\mu_{it}$ and $\pi_{it}$ are the error terms. The subscript *i* = 1, …, *N* represents the country, and *t* = 1, …, *T* represents the period.

We constructed the simultaneous Equations (3)–(5) for analyzing the association between CE, EG, and FDI by incorporating the important economic variables. Equation (3) postulates that carbon emissions (CE) can be potentially affected by economic growth (EG), FDI, financial development (FD), urban population (UP), energy consumption (EC) (e.g., Hossain [48], Lotfalipour et al. [44], and Shahbaz et al. [17]. Equation (4) demonstrates that carbon emissions (CE), FDI, domestic capital (DS), inflation rate (INF), and labor force (LF) are the possible determining factors of economic growth (EG) (e.g., Anwar and Sun [60]; Lin and Nelson [58]). Equation (5) implies that economic growth (EG), carbon emissions (CE), labor force (LF), the exchange rate (EX), domestic capital (DS), and trade openness (TD) are possible determining factors of FDI (e.g., Anwar and Sun [60], Lin and Nelson [58]). Assuming that all variables follow the unit root process, while error terms are stationary ($\eta_{it}$ ~ *I*(1)), the Equation (3) represents the panel co-integration with panel vector error correction model (PVECM) as follows:

For VECM:

(6) $$\Delta lnCO_{2,it}=\varphi_{1}ecm_{i,t-1}+\delta_{11}\Delta lnGDPP_{it}+\delta_{21}\Delta lnFDI_{it}+\delta_{31}\Delta lnFD_{it}+\delta_{41}\Delta lnUP_{it}+\delta_{51}\Delta lnEC_{it}+\in_{1it}$$

(7) $$\Delta lnGDPP_{it}=\varphi_{2}ecm_{i,t-1}+\delta_{12}\Delta lnCO_{2,it}+\delta_{22}\Delta lnFDI_{it}+\delta_{32}\Delta lnFD_{it}+\delta_{42}\Delta lnUP_{it}+\delta_{52}\Delta lnEC_{it}+\in_{2it}$$

(8) $$\Delta lnFDI_{it}=\varphi_{3}ecm_{i,t-1}+\delta_{13}\Delta lnCO_{2,it}+\delta_{23}\Delta lnGDPP_{it}+\delta_{33}\Delta lnFD_{it}+\delta_{43}\Delta lnUP_{it}+\delta_{53}\Delta lnEC_{it}+\in_{3it}.\;$$

(9) $$\Delta lnFD_{it}=\varphi_{4}ecm_{i,t-1}+\delta_{14}\Delta lnCO_{2,it}+\delta_{24}\Delta lnGDPP_{it}+\delta_{34}\Delta lnFDI_{it}+\delta_{44}\Delta lnUP_{it}+\delta_{54}\Delta lnEC_{it}+\in_{4it}$$

(10) $$\Delta lnUP_{it}=\varphi_{5}ecm_{i,t-1}+\delta_{15}\Delta lnCO_{2,it}+\delta_{25}\Delta lnGDPP_{it}+\delta_{35}\Delta lnFDI_{it}+\delta_{45}\Delta lnFD_{it}+\delta_{55}\Delta lnEC_{it}+\in_{5it}$$

(11) $$\Delta lnEC_{it\;}=\varphi_{6}ecm_{i,t-1}+\delta_{16}\Delta lnCO_{2,it}+\delta_{26}\Delta lnGDPP_{it}+\delta_{36}\Delta lnFDI_{it}+\delta_{46}\Delta lnFD_{it}+\delta_{56}\Delta lnUP_{it}+\in_{6it}$$

From Equations (6)–(11), $ecm_{i,t-1}$ represents the error correction term; $\varphi_{1}$, $\varphi_{2}$, $\varphi_{3}$, $\varphi_{4}$, $\varphi_{5}$, and $\varphi_{6}$ capture the long-term equilibrium association between variables. ∆s are the difference operators. $\varphi_{1}<0$, $\varphi_{2}<0$, $\varphi_{3}<$, $\varphi_{4}<0$, $\varphi_{5}<0$, and $\varphi_{6}<0$ postulate that long-term association does not obstruct fluctuations in CO_2_, GDPP, FDI, financial development, urban population, and energy consumption, while the greater sign demonstrates the opposite meaning. $\in_{1t}$, $\in_{2t}$, $\in_{3t}$, and $\in_{4t}$ are the error terms.

### 3.4. Dynamic Ordinary Least Squares (DOLS) and Co-Integration Tests

There are two prominent methods to find consistent estimators in panel models, dynamic ordinary least squares (DOLS) and fully modified ordinary least squares (FMOLS). However, the DOLS is one of the best estimators particularly in the finite samples as compared to other alternative methods. It helps to control the issue of endogeneity and serial correlation biases in the model and provides robust results about the relationship among variables [61,62]. It allows the integration of variables on different orders and deals with the problem of simultaneity among regressors [63]. The DOLS method is preferred over the FMOLS method because it performs better [62]. According to Wagner and Hlouskova [64], DOLS performs better as compared to others methods in both single and system of equations estimators, even for large samples. According to Harris and Sollis [65], DOLS provides more robust results as compared to FMOLS. Therefore, we utilized panel DOLS to find prominent features in the economic outlook and long-run association in the panel for selected countries.

In this study, we employed the DOLS along with Engle-Granger based co-integration tests (Pedroni and Kao methods and Fisher combined Johansen co-integration test) to explore co-integration in the simultaneous equations of CE, EG, and FDI as a group and individual countries in line with prior studies conducted by Mitić et al. [61], Lin and Nelson [58], and Ouedraogo [66]. The summary of panel DOLS for Equation (3) is given below:

$$u_{it}=lnC02_{it}\;\text{be a scalar}\;v_{it}=\left(lnGDPP_{it},\;lnFDI_{it},\;lnFD_{it},\;lnUP_{it},\;lnEC_{it}\right)$$

If is an n-dimensional vector then ${\left(u_{it},\;v_{it}^{\prime }\right)}^{\prime }$ is a (n+1) dimensional vector of variables that fulfills the conditions as follows:

(12) $$u_{it}=\xi_{i}+\omega_{it}+\phi_{t}+\kappa^{\prime }v_{it}+\eta_{it}^{*}$$

Assume that $\left(1,-\kappa^{\prime }\right)$ is a cointegration vector, $u_{it}-\kappa^{\prime }v_{it}$ is a composite equilibrium error that includes $\xi_{i}$(individual-specific), $\omega_{it}$(linear-trend) and $\phi_{t}$ (time-specific), $\eta_{it}^{*}$ is the idiosyncratic error term.

If we put $\omega_{i}=0$ and $\phi_{t}=0\;\forall \;t$ and t in (12), we have:

(13) $$u_{it}=\xi_{i}+\kappa^{\prime }v_{it}+\eta_{it}^{*}$$

To overcome the endogeneity bias that might occur in case $\eta_{it}$ is associated with at most $P_{i}$ leads and lags of $q_{it}=\Delta v_{it}$, we proposed $\eta_{it}$ and attained as follows:

(14) $$\eta_{it}^{*}=\overset{p_{i}}{\underset{r=-p_{i}}{\sum }}\chi_{i,\;s}^{\prime }q_{it-s}+\eta_{it}=\overset{p_{i}}{\underset{s=-p_{i}}{\sum }}\chi_{i,\;s}^{\prime }\Delta v_{it-s}+\eta_{it}=\chi_{i}^{\prime }\;g_{it}+\eta_{it}$$

where $\chi_{i,\;s}$ is the projection coefficient, $\chi_{i}={\left(\chi_{i,-p_{i}}^{\prime },\ldots ,\chi_{i,\;0}^{\prime }\ldots ,\chi_{p_{i}}^{\prime }\right)}^{\prime }$ is a vector with $\left(2p_{i}+1\right)n$ dimensions and $g_{it}={\left(\Delta v_{it,-p_{i}}^{\prime },\ldots ,\Delta v_{it}^{\prime }\ldots ,\Delta v_{it+p_{i}}^{\prime }\right)}^{\prime }$ is a vector of leads and lags. Replacing the orthogonal projection of $\eta_{it}$ denoted in Equation (14) into Equation (13), we have:

(15) $$u_{it}=\xi_{i}+\kappa^{\prime }v_{it}+\chi_{i}^{\prime }\;g_{it}+\eta_{it}$$

The covariance-stationary vector process represented as $\tau_{it}={\left(\eta_{it},\;q_{it}^{\prime }\right)}^{\prime }$, $\tau_{it}=\Omega_{i}\left(L\right)\varepsilon_{it}$ and $\Omega_{i}\left(L\right)=\left|\begin{array}{c} \begin{array}{cc} \Omega_{uu,i}\left(L\right) & O^{\prime } \end{array}\\ \begin{array}{cc} O & \Omega_{uu,i} \end{array} \end{array}\right|$ where $\tau_{it}$ follows the central limit theorem $\frac{1}{\sqrt{T}}\;\sum_{t=1}^{T_{r}}\tau_{it}\overset{D}{\to }A_{i}=\Omega_{i}\left(1\right)W_{i}$, where $A_{i}={\left(A_{ui},\;A_{vi}^{\prime }\right)}^{\prime }$ are independent and $\Gamma_{i}=\left[A_{i}\left(1\right)A_{i}{\left(1\right)}^{\prime }\right]=\left[\begin{array}{c} \Omega_{uu,i}{\left(1\right)}^{2}\;O^{\prime }\\ \;0\;\Omega_{vv,i}\left(1\right)\Omega_{vv,i}{\left(1\right)}^{\prime } \end{array}\right]=\left[\begin{array}{c} \begin{array}{cc} \Gamma_{uu,i} & O \end{array}\\ \begin{array}{cc} O & \Gamma_{vv,i} \end{array} \end{array}\right]$. The average function of Equation (15) is as follows:

(16) $$\frac{1}{T}\;\overset{T}{\underset{t=1}{\sum }}u_{it}=\xi_{i}+\kappa^{\prime }\frac{1}{T}\;\overset{T}{\underset{t=1}{\sum }}v_{it}+\chi_{i}^{\prime }\frac{1}{T}\;\overset{T}{\underset{t=1}{\sum }}g_{it}+\frac{1}{T}\;\overset{T}{\underset{t=1}{\sum }}\eta_{it}$$

By subtracting the Equation (16) from the Equation (15), we have:

(17) $${\tilde{u}}_{it}=\kappa^{\prime }{\tilde{v}}_{it}+\chi_{i}^{\prime }\;{\tilde{g}}_{it}+{\tilde{\eta }}_{it}$$

where ${\tilde{u}}_{it}=u_{it}-\frac{1}{T}\;\sum_{t=1}^{T\;}u_{it}$, ${\tilde{v}}_{it}=v_{it}-\frac{1}{T}\;\sum_{t=1}^{T}v_{it}$, ${\tilde{g}}_{it}=g_{it}-\frac{1}{T}\;\sum_{t=1}^{T}g_{it}$, ${\tilde{\eta }}_{it}=\eta_{it}-\frac{1}{T}\;\sum_{t=1}^{T}\eta_{it}$. Lastly, $R_{it}$ be a vector with $2n\left(1+\sum_{i=1}^{N}\sum p_{i}\right)$ dimension whose primary n components are ${\tilde{v}}_{it}$, $n\left\{\sum_{j=1}^{i-1}\left(1+2p_{j}\right)+1\right\}+1$ to $n\left\{\sum_{j=1}^{i}\left(1+2p_{j}\right)+1\right\}$ are ${\tilde{g}}_{it}$ and zeros, inferring:

${\tilde{R}}_{1t}={\left({\tilde{v}}_{1t}^{\prime }\;{\tilde{g}}_{1t}^{\prime }\;O^{\prime }\;\ldots \;O^{\prime }\right)}^{\prime }$
${\tilde{R}}_{2t}={\left({\tilde{v}}_{1t}^{\prime }\;O^{\prime }\;{\tilde{g}}_{2t}^{\prime }\;\ldots O^{\prime }\right)}^{\prime }$
⋮    ⋮
${\tilde{R}}_{Nt}={\left({\tilde{v}}_{Nt}^{\prime }\;O^{\prime }\;O^{\prime }\;\ldots \;{\tilde{g}}_{Nt}^{\prime }\right)}^{\prime }$

The vector of a coefficient is $\sigma ={\left(\kappa^{\prime },\;\chi_{1}^{\prime },\ldots ,\chi_{N}^{\prime }\right)}^{\prime }$, and the compressed regression equation is ${\tilde{u}}_{it}=\sigma^{\prime }{\tilde{R}}_{it}+{\tilde{\eta }}_{it}$. Finally, the panel dynamic ordinary least squares estimator is $\sigma_{NT}$, where

(18) $$\sigma_{NT}-\sigma ={\left[\overset{N}{\underset{i=1}{\sum }}\overset{T}{\underset{t=1}{\sum }}{\tilde{R}}_{it}{\tilde{R}}_{it}^{\prime }\right]}^{-1}\left[\overset{N}{\underset{i=1}{\sum }}\overset{T}{\underset{t=1}{\sum }}{\tilde{R}}_{it}{\tilde{\eta }}_{it}\right]$$

We explored that ${\tilde{\eta }}_{it}$ is identical to $\eta_{it}$ and algebra also exposes the following setting: $\frac{1}{T}\;\sum_{t=1}^{T\;}{\tilde{v}}_{it}{\tilde{\eta }}_{it}=\frac{1}{T}\;\sum_{t=1}^{T\;}{\tilde{v}}_{it}\eta_{it}\overset{D}{\to }\sqrt{\Gamma_{uu,i}}\int {\tilde{A}}_{vi}dW_{ui}$, where ${\tilde{A}}_{vi}=A_{vi}-\int A_{vi}$. Information about variables measurement and data sources is given in Table 1.

## 4. Results and Discussion

### 4.1. Summary Statistics

Table 2 highlights the summary statistics for each variable to examine the generalized view of CO_2_, GDPP, FDI, and other variables for the entire panel dataset.

### 4.2. Unit Root Test

To test the stationarity of variables for a group of countries, we employed different panel unit root tests. Table 3 presents the results of these tests, which help us to decide whether the series of variables are stationary or non-stationary series based on probability values. The results of all these tests- LL, IPS, Fisher-ADF, and Fisher-PP validated that all variables used in this study are shown non-stationary at level, but the variables are converted into stationary form by taking the first difference. These results fulfill the stationary property for each variable that provides a strong basis for panel co-integration analysis. It is required because applying the regression on non-stationary series can give spurious results.

### 4.3. Dynamic Ordinary Least Squares (DOLS) and Co-integration Test

We employed DOLS to find a long-run association between CO_2_, GDPP, and FDI along with other economic determinants for the group and individual countries. Table 4 presents the results for the group of countries. The results of Model (3) reveal that GDPP significantly positively affects CO_2_ at 10% level. This result implies that an increase in GDPP by 1 unit increases CO_2_ by 0.3188 units. Theoretically, the increase in production level may increase the economic growth (EG), and when the production level increases at an intense level, it pollutes the environment. This result corresponds to the prior studies conducted by Pandey and Mishra [51], and Jaunky [67]. From the controlling factors, the results show that urban population (UP) and energy consumption (EC) significantly negatively affect CO_2_ in the SAARC countries (see Table 4). The results of model (4) demonstrate that the coefficients of both key variables including CO_2_ and FDI are statistically insignificant. The other determining factors of GDPP including domestic capital (DS) and inflation rate (INF) have significantly positive effects on GDPP. In SAARC countries, labor market is quite attractive and cheap, which progressively contributes to the economy of these countries. In SAARC countries, the flow of domestic capital along with foreign capital is higher, which helps to grow the overall economy of this region. The results of model (5) show that the coefficients of both CO_2_ and GDPP are statistically insignificant. Among the controlling factors, domestic capital (DS) significantly positively affects FDI in the SAARC countries. Hence, the results of these three models endorse the unidirectional causal association of economic growth (EG) with carbon emission (CE) for SAARC countries. This finding validates the findings of prior studies conducted by Abdouli and Hammami [28], Jaunky [67] and Lotfalipour et al. [44] (see Table 4).

Table 5 and Table 6 demonstrate the results for individual countries. The results of Model (3) for Bangladesh reveal that GDPP significantly positively affects CO_2_ at 1% level. It proposes that an increase in GDPP by 1 unit can augment the CO_2_ by 0.7196 units. Moreover, FDI significantly negatively affects CO_2_ at 5% level. It advocates that an increase in FDI by 1 unit decreases the CO_2_ by 0.024 units. Attaining the environmentally sound development like developed countries was an emerging issue for Bangladesh in recent years, therefore, Bangladesh adopted the policies to deal with environmental challenges, and accomplished numerous milestones in the environment sector despite the difficulties of poverty, overpopulation, corruption, and lack of resources [68]. Among the controlling factors, financial development (FD) and urban population (UP) have significant positive effects on CO_2_ at 1% level, while EC significantly negatively affects CO_2_ at 1% level. The results of Model (4) for Bangladesh show that the coefficients of both main key factors including CO_2_ and FDI are statistically insignificant. From the controlling factors, the results reveal that inflation significantly positively influences CO_2_ at 10% level. The results of Model 5 imply that CO_2_ significantly negatively affects FDI at 5% level. It implies that the addition to CO_2_ by 1 unit decreases the FDI by 4.3554 units. Moreover, the results reveal that GDPP significantly positively affects the FDI at 1% level, it advocates that an augmentation in the GDPP by 1 unit increases the FDI by 5.0376 units. Among the controlling factors, domestic capital (DS) significantly positively influences FDI at 1% level, while labor force (LF) and exchange rate (EX) have negative effects on FDI at 5% level. Finally, the results of these models confirm the unidirectional causal association of economic growth (EG) with carbon emissions (CE), the bidirectional causal association between FDI and CE, and unidirectional causality from EG to FDI. These findings are in line with previous studies by Jaunky [67], and Omri, Nguyen and Rault [35] (See Table 5).

The results of Model (3) for India show that GDPP has a significant positive effect on CO_2_ at 5% level. It suggests that an increase in GDPP by 1 unit increases the CO_2_ by 0.7386 units. Moreover, the results highlight that FDI significantly negatively affects CO2 at 10% level. It postulates that addition to FDI by 1 unit can reduce the CO_2_ by 0.057 units. India endorsed the Paris agreement and took measures for environmental protection during the growth process. In recent years, India has adopted policies to expand its renewable power, especially solar with the help of foreign investors [69]. Among the controlling factors, EC significantly negatively influences CO_2_ at 10% level. The results of Model (4) for India reveal that CO_2_ significantly positively affects GDPP at 1% level. It postulates that an augmentation in CO_2_ by 1 unit increases the GDPP by 2.6166 units. Among the controlling factors, domestic capital (DS) and labor force (LF) significantly positively affect GDPP at 1% level, while inflation (INF) has a significant negative effect on GDPP at 10% level. The results of Model (5) for India indicate that CO_2_ significantly positively affects the FDI at 5% level. Among the controlling factors, labor force (LF) significantly positively influences FDI at 1% level. Finally, the results of these models confirm bidirectional causal association between EG and CE, bidirectional causal association between FDI and CE, and unidirectional causality from EG to FDI. These findings are similar to prior researches conducted by Pao and Tsai [70], and Olusanya [71] (See Table 5).

The results of Model (3) for Nepal show that the coefficients of both GDPP and FDI are statistically insignificant, which suggests that there is no significant effect of these variables on CO_2_. Nepal is one of the richest countries ecologically, but poor economically [72]. Nepal has also signed the Paris agreement and made international commitments for attaining economic, social, and environmental growth in the future. In light of the Paris agreement, Nepal has attained almost six-fold progress in terms of green growth by following the sustainable development goals (SDGs) to achieve the status of a least developed country (LDC) in recent years [73]. Among the controlling factors, financial development (FD) has a significant positive effect on CO_2_ at 5% level, while EC has a significant negative effect on CO_2_ at 1% level. The results of Model (4) for Nepal demonstrate that the coefficients of both CO_2_ and FDI are statistically insignificant, which infer that there is no significant effect of these variables on GDPP. Among the controlling factors, inflation has a significant positive effect on GDPP at 10% level. The results of Model (5) for Nepal reveal that the coefficients of key variables including CO_2_, GDPP, and other variables are statistically insignificant. Finally, the results of these models confirm that there is no causal association between key variables including economic growth (EG), FDI and carbon emission (CE). This result correlates with the findings of a previous study conducted by Shaari, et al. [74] (See Table 6).

The results of Model (3) for Pakistan reveal that the coefficients of both FDI and GDPP are statistically insignificant, which implies that there is no significant effect of these variables on CO_2_. During the recent few decades, the economy of Pakistan has demonstrated enormous growth and great potential for growth in the future. Pakistan has persistently got benefits from globalization in terms of trade, as a result, the energy demand was increased in Pakistan in recent years. However, the economic development created environmental challenges for Pakistan [75]. To deal with these environmental challenges, Pakistan initially formulated an environmental policy in 2005 [76]. Pakistan as a developing and emerging economy is the major destination for foreign investment, while foreign investment is the major source for technology transfer [77]. At present, Pakistan is trying its best for technology transfer under the umbrella of foreign investment, particularly through China Pakistan Economic Corridor (CPEC) project to deal with environmental challenges [78]. Among the controlling factors, urban population (UP) and energy consumption (EC) have significant negative effects on CO_2_ at 1% and 10% levels respectively. The results of Model (4) for Pakistan indicate that the coefficients of both CO_2_ and FDI are statistically insignificant, which reveals that there is no significant effect of these variables on GDPP. Among the controlling factors, domestic capital (DS) and inflation (INF) have significant positive effects on GDPP at 10% and 1% levels respectively. The results of Model (5) for Pakistan reveal that CO_2_ has a significant positive association with FDI at 1% level. It infers that an increase in CO_2_ by 1 unit increases the FDI by 10.266 units. Furthermore, the results indicate that GDPP significantly negatively affects FDI at 10% level. It infers that the addition to GDPP by 1 unit decreases the FDI by 1.3627 units in Pakistan. Among the controlling factors, domestic capital (DS), labor force (LF), the exchange rate (EX), and trade openness (TD) have significant positive effects on FDI at different significance levels. Finally, the results of these models confirm the unidirectional causality from carbon emissions (CE) and economic growth (EG) to FDI. These results are in line with a study conducted by Olusanya [71] (See Table 6).

The results of Model (3) for Sri Lanka demonstrate that GDPP significantly positively affects CO_2_ at 1% level. It infers that addition to GDPP by 1 unit can augment the CO_2_ by 0.8364 units. Furthermore, FDI significantly negatively associates with CO_2_ at 5% level. It postulates that addition to FDI by 1 unit decreases CO_2_ by 0.5519 units. In the early years, Sir Lanka like other developing countries was failed to attain substantial progress to controlling the risk of climate changes [8]. However, Sri Lanka adopted the comprehensive national action plan to deal with climate change challenges in 2015. These policy-level initiatives have yet to implement in true letter and spirit because of lack of stakeholders support, insufficient policy level directions, and public awareness [79]. Among the other economic factors, FD significantly positively influences CO_2_ at 5% level. The results of Model (4) for Sri Lanka indicate that the coefficients of both CO_2_ and FDI are statistically insignificant, which infer that both CO_2_ and FDI have no significant effect on GDPP. Among the controlling factors, domestic capital (DS) and inflation (INF) are significantly positively associated with GDPP at 10% and 1% levels respectively. The results of Model (5) indicate that GDPP significantly positively affects FDI at 10% level. It postulates that the addition to GDPP by 1 unit increases the FDI by 1.4789 units. Among the controlling factors, trade (TD) significantly positively influences FDI at 5% level. Finally, the results of these models confirm the unidirectional causality from economic growth (EG) and FDI to carbon emissions (CE), and the unidirectional causal association of EG with FDI. These results correlate with the study conducted by Lee [34] (See Table 6).

In addition to DOLS, we employed the panel co-integration tests together with the Pedroni and Kao methods to observe the co-integration among the variables considered in the models of CO_2_, GDPP, and FDI, the results are shown in Table 7. The number of lags is chosen in line with the Akaike information criterion (AIC). The results for panel co-integration tests in Model (3) reveal that panel PP-statistics validate the presence of co-integration among variables considered in the model of CO_2_ at 1% level, while panel ADF-statistics also confirm the co-integration at 1% level. Moreover, group PP-statistics, group ADF-statistics, and ADF t-statistics combined with the Kao method also confirm the co-integration among variables at 1% level.

In model (4), panel ADF-statistics, group ADF-statistics, and ADF t-statistics validate the presence of long-run connections among variables at 5% and 1% levels respectively. In model (5), panel PP-statistics, panel ADF statistics, group PP-statistics, group ADF-statistics, and ADF t-statistics endorse the co-integration among variables at 1% level. Hence, it can be concluded that there is a long-run co-integration among variables considered in models of carbon emissions (CE), economic growth (EG), and FDI.

### 4.4. Vector Error Correction Model (VECM)

After finding the co-integration between variables, we employed VECM for a group of SAARC countries to determine the directions of causal association among variables considered in the main model (3) on carbon emissions (CO_2_). The results of model (6) demonstrate that the coefficient of $ecm_{t-1}$ is statistically insignificant in the equation of CO_2_. Moreover, FDI, financial development (FD), and urban population (UP) induce short-run dynamic connection with CO_2_ at 1% significance level. The results of model (7) show that the coefficient of $ecm_{t-2}$ is significant in the equation of GDPP (i.e., $ecm_{t-2}$ is 0.0119). It postulates that the speed of adjustment of GDPP is 1.19% towards long-run equilibrium. The results of Model (8) indicate that coefficients of all variables are statistically insignificant including $ecm_{t-3}$ (see Table 8).

The results of Model (9) indicate that the coefficient of $ecm_{t-4}$ is significant in the equation of financial development (FD) (i.e., $ecm_{t-4}$ is −0.0352). It implies that the speed of adjustment of FD is 3.52% towards the long-run equilibrium. The results of Model (10) show that the coefficient of $ecm_{t-5}$ is significant in the equation of urban population (UP) (i.e., $ecm_{t-5}$ is 0.1035). It postulates that the speed of adjustment of UP is 10.35% towards long-run equilibrium. Furthermore, financial development (FD) and EC induce the short-run dynamic connection with the urban population (UP) at different significance levels. The results of Model (11) reveal that the coefficient of $ecm_{t-6}$ is significant in the equation of EC with a positive sign ($ecm_{t-6}$ is 0.0093). It postulates that the speed of adjustment of EC is 0.93% towards long-run equilibrium. Furthermore, financial development (FD) and urban population (UP) induce short-run dynamic connection with EC at 1% level (see Table 8).

### 4.5. Impulse Response Analysis (IRA)

**Figure 1:**
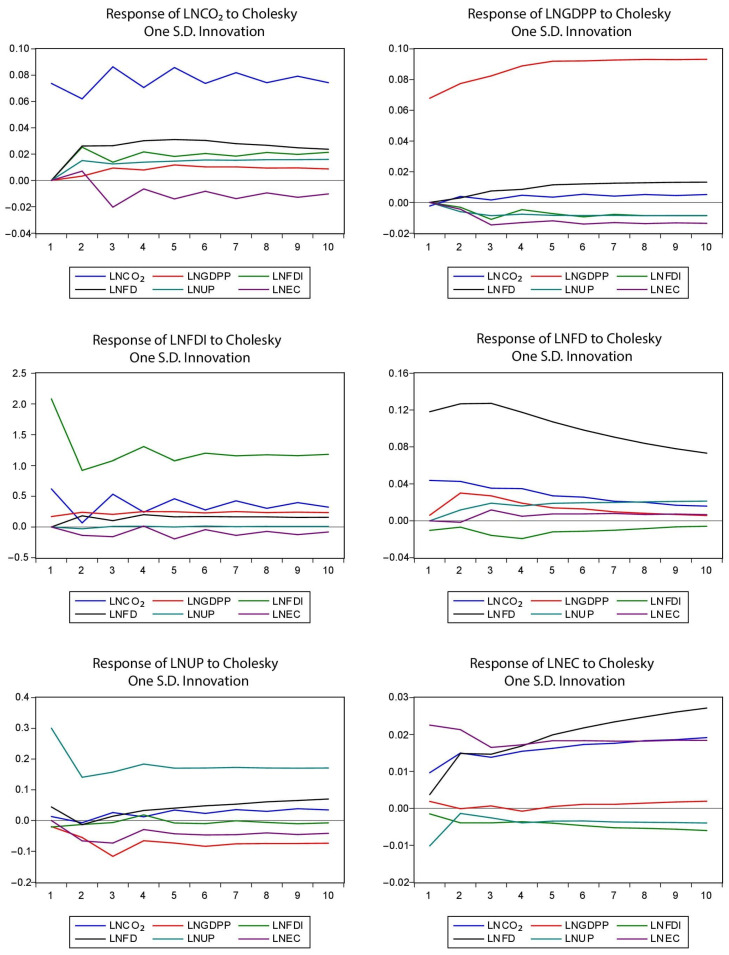
Impulse response analysis.

We drafted the IRA in the order of variables with a time horizon of 10 years period. The dependent variable is prioritized as the first variable in the order of variables, and others are the explanatory variables. Figure 1 shows that shocks in all explanatory variables including EG, financial development (FD), FDI, and urban population (UP) have a positive association with carbon emission (CO_2_) over the period. Contrarily, EC has a positive association with CO_2_ in the initial two years, while it has a negative connection with CO_2_ in all other years for the SAARC countries.

Figure 1 also reveals that shocks in the CO_2_ and financial development (FD) have positive shocks to GDPP, while other variables have negative shocks to GDPP over the period for the SAARC countries. Figure 1 also depicts that the shocks in CO_2_, financial development (FD), and GDPP have positive shocks to FDI over the ten years, while the shocks in energy consumption (EC) have a negative effect over the period for the SAARC countries.

Figure 1 also demonstrates that the shocks in CO_2_, GDPP, urban population (UP) have positive shocks to financial development (FD), while shocks in energy consumption (EC) have negative effects on FD in the initial two years, afterwards, these shocks have positive effects on FD in the rest of period. Moreover, shocks in FDI have negative effects on FD over the period for the SAARC countries.

Figure 1 also reveals that shocks in CO_2_ and FD have a positive association with the urban population (UP) in the whole period except in the second year. Furthermore, shocks in FDI have a mixed trend of effects on UP for the whole period. Contrarily, shocks in GDPP and EC positively influenced UP over the period for the SAARC countries.

Figure 1 also depicts that shocks in CO_2_ and FD have a positive association with energy consumption (EC) in the whole period. Moreover, shocks in GDPP have negative effects on EC from second to fifth years, afterwards, these have positive effects on EC in the rest of the period. Furthermore, shocks in FDI and urban population (UP) have negative effects on EC over the period for the SAARC countries.

### 4.6. Variance Decomposition Analysis (VDA)

Table 9 shows the results of the VDA of carbon emission. Results suggest that carbon emission (CO_2_) has a robust self-explanatory influence in the short-run, whereas it is reduced to 80% in the long run. Contrarily, other variables including EG, FDI, financial development (FD), urban population (UP), and energy consumption (EC) have no shocks to CO_2_ in the short run. Furthermore, 1.05% of GDPP is described by shocks to CO_2_ in the long run. Moreover, CO_2_ is affected by 5.05% shocks of FDI in the long run. Additionally, 9.36% of financial development (FD) is described by shocks to CO_2_ in the long run. Moreover, 2.76% of the urban population (UP) is explained by shocks to CO_2_ in the long run. Likewise, CO_2_ is affected by 1.8% of energy consumption (EC) shocks in the long run.

## 5. Conclusions and Policy Implications

In this study, we empirically analyzed the dynamic causal interaction among FDI, economic growth (EG), carbon emission (CE) and other economic factors for the group and individual SAARC countries by employing the panel dynamic ordinary least squares (DOLS) and panel cointegration for the period 1990 to 2016. The following economic factors have been considered as control variables in this study- financial development (FD), urban population (UP), domestic capital (DS), energy consumption (EC), labor force (LF), exchange rate (EX), inflation rate (INF), and trade openness (TD).

The results for the group of countries confirmed the existence of co-integration among variables in all empirical models. The results estimated by DOLS confirmed the presence of the unidirectional causal association of economic growth (EG) with carbon emission (CE). These results are in line with the prior studies conducted by Abdouli and Hammami [28], Jaunky [67] and Lotfalipour et al. [44]. For instance, Abdouli and Hammami [28] revealed the unidirectional causality from EG to CE in the case of Jordan, Kuwait, Oman, and Tunisia by studying the sample period 1990–2012. Moreover, Jaunky [67] demonstrated the unidirectional causality from EG to CE in the case of Greece, Malta, Oman, Portugal and the United Kingdom by selecting the sample period 1980–2005. Furthermore, Lotfalipour et al. [44] also revealed the unidirectional causal association of EG with CE in Iran by using the data for the period 1967 to 2007.

The results of our study revealed a positive association between economic growth (EG) and carbon emission (CE). These results validate the environmental Kuznets curve (EKC). According to EKC, there is a strong positive association between the environment and economic development during the industrialization process. EG is the main cause of increased environmental degradation, but after a certain point of time, the association follows an inverted U shape trend [41]. The inverted U-shaped association between environment and EG is based on three types of effects such as scale effect, composition effect, and technique effect, while, the composition effect suggests the positive association of EG with the environment [40]. The results of the control variables confirmed the causality of urban population (UP) and energy consumption (EC) with carbon emissions (CE). Moreover, domestic capital (DS) and inflation rate (INF) confirmed the causal association with EG. Besides, the result confirmed the causality of domestic capital (DS) with FDI.

The empirical results for individual countries confirmed the unidirectional causal association of EG with CE, the bidirectional causal association between FDI and CE, and unidirectional causality from EG to FDI for Bangladesh. In the case of India, the results validated the bidirectional causal association between EG and CE, the bidirectional causal association between FDI and CE, and unidirectional causality from EG to FDI. In the case of Nepal, the results revealed that there is no causal association among the variables- EG, FDI, and carbon emission (CE). In the context of Pakistan, the results revealed the presence of unidirectional causality from CE and EG to FDI. In the case of Sri Lanka, the results validated the unidirectional causality from EG and FDI to CE, and the unidirectional causal association of EG with FDI. These results are consistent with studies conducted in other developed and developing countries by Lee [34], Omri et al. [35], Pao and Tsai [70], Feridun and Sissoko [80], and Sebri and Ben-Salha [50]. The results of the panel vector error correction model (PVECM) confirmed the long-run equilibrium relationship in the equations of GDPP, financial development (FD), urban population (UP), and energy consumption (EC). Furthermore, the results established the short-run dynamic relationship estimated by VECM from FDI, FD, and UP to CE; economic growth (EG) to FD; FD and EC to UP; and FD and UP to EC.

Based on the results, we can conclude that economic growth (EG) significantly contributes to carbon emission (CE) in the SAARC countries. There are several causes of polluting the environment of the South Asian region such as water pollution, wastage, air pollution, urbanization, etc., however, CO_2_ emission is the main cause of polluting the environment of this region [14]. CO_2_ emission is one of the main concentrations of greenhouse gases (GHGs) [81] and it is consistently being increased over time in this region. The main causes of increasing CO_2_ emissions in this region include rapid industrialization, urban population, and the burning of fossil fuels. Pakistan and India are the main contributors to the pollution in South Asia since they produce the highest level of CO_2_ emissions among all of the SAARC countries. It is a major threat to the security of billions of people living in this region [14,82].

### 5.1. Policy Implications

From the environmental perspective of the study, it is suggested that developing countries, in particular the SAARC countries, should formulate strict environmental regulations in the growth process for the environmental protection of the region. Developing countries, in particular the SAARC countries, should set strict qualification criteria for foreign investors to evade environmental destruction. Investment-oriented and trade liberalization policies should be adopted in developing countries, particularly in the SAARC countries. By increasing the foreign investment, trade, and green technology can be transferred to the host countries. Moreover, efforts should be made to promote the usage of green technology in developing countries, particularly in the SAARC countries, for decreasing carbon emissions (CE). Furthermore, policymakers in the SAARC countries, particularly in Pakistan and India, should formulate policies to control the population ratio, especially in urban areas. Moreover, they should formulate policies to discourage the usage of fossil fuels and promote alternatives for controlling the CE in the region.

### 5.2. Limitations and Future Research

This study is limited to the specific period from 1990 to 2016, and specific economic variables due to data constraints. Future researchers can extend the study period and consider institutional and social factors from environmental perspectives. Moreover, it is suggested for future research that studying the role of financial development from an energy perspective and incorporating the bond and stock market-related variables of developing countries, could be interesting and a significant contribution to the empirical literature. There is a new study conducted by Shahbaz, Nasir and Roubaud [17] which examines the environmental degradation of France through the use of the newly developed technique- bootstrapping bounds test developed by McNown, et al. [83]. It is recommended that future studies may test the environmental Kuznets curve (EKC) in developing countries by employing the bootstrapping bounds testing approach.

## Figures and Tables

**Table 1:** Variables Measurement.

Variables	Measures	Data Source
**CO_2_**	Carbon Emissions (CE)-carbon dioxide emissions (metric tons per capita)	WDI-database [54]
**GDPP**	Economic growth (EG) -GDP per capita (Current USD)	WDI-database [54]
**FDI**	Net FDI inflows (Current USD)	WDI-database [54]
**FD**	Financial development-domestic credit to the private sector (% of GDP)	WDI-database [54]
**UP**	Urban population growth (annual %)	WDI-database [54]
**EC**	Energy consumption-Kg of Oil equivalent (Per Capita)	SAARC energy outlook 2030 [55]
**DS**	Domestic capital-Gross fixed capital formation (% of GDP)	WDI-database [54]
**LF**	Labor force-total	WDI-database [54]
**INF**	Inflation rate-consumer price index (CPI)	IMF-Database [56]
**EX**	Real foreign exchange rate (CPI- Based)	Bruegel Datasets [54]
**TD**	Trade openness-total trade (% of GDP)	WDI database [54]

**Table 2:** Summary statistics.

Variables	Observations	Mean	Std. Dev.	Median	Maximum	Minimum
CO_2_	135	0.5879	0.4096	0.5800	1.8178	0.0411
GDPP	135	831.3459	752.1110	576.1956	3886.2920	170.5867
FDI	135	3,560,000,000	9,220,000,000	430,000,000	44,500,000,000	(6,647,984)
FD	135	30.3810	12.9016	27.8432	80.8453	8.8212
UP	135	2.9544	1.5447	2.8517	6.9946	0.0466
EC	135	374.9077	127.8014	397.3782	698.1262	118.8983
DS	135	22.9357	5.3319	23.3589	35.8129	12.5206
LF	135	106,000,000	157,000,000	38,981,417	481,000,000	7,018,188
INF	135	69.6049	39.5012	57.2523	165.7844	14.4872
EX	135	103.9490	14.8973	100.2830	161.1529	78.8423
TD	135	43.3280	16.9629	41.8283	88.6364	15.5063

**Table 3:** Results of Unit Root Tests (Panel).

Variables	LL	IPS	Fisher-ADF	Fisher-PP
LNCO_2_	0.3515 (0.6374)	2.502 (0.9938)	1.6496 (0.9984)	2.0242 (0.9962)
LNGDPP	3.4990 (0.9998)	6.0949 (1.0000)	0.2381 (1.0000)	0.2314 (1.0000)
LNFDI	−0.8357 (0.2017)	0.1535 (0.5610)	6.6403 (0.7589)	18.789 (0.0430)
LNFD	−0.4205 (0.3370)	1.4886 (0.9317)	3.1714 (0.9771)	2.5445 (0.9902)
LNUP	−0.1333 (0.4470)	0.4193 (0.6625)	7.3714 (0.6900)	16.102 (0.0967)
LNEC	3.7076 (0.9999)	5.2052 (1.0000)	3.9979 (0.9474)	3.7800 (0.9567)
LNDS	−2.6226 (0.0044)	−1.1765 (0.1197)	15.661 (0.1098)	11.257 (0.3379)
LNLF	−2.0809 (0.0187)	1.3434 (0.9104)	6.6145 (0.7613)	54.005 (0.0000)
LNINF	−0.3049 (0.3802)	2.7297 (0.9968)	3.6326 (0.9624)	3.9681 (0.9488)
LNEX	1.8693 (0.9692)	2.1580 (0.9845)	1.9006 (0.9970)	2.1681 (2.1681)
LNTD	−0.7786 (0.2181)	−0.1727 (0.4315)	9.1453 (0.5184)	11.049 (0.3537)
∆LNCO_2_	−7.2154 (0.0000)	−10.002 (0.0000)	90.410 (0.0000)	97.499 (0.0000)
∆LNGDPP	−7.6346 (0.0000)	−7.0005 (0.0000)	60.836 (0.0000)	61.379 (0.0000)
∆LNFDI	−10.898 (0.0000)	−11.212 (0.0000)	99.789 (0.0000)	97.135 (0.0000)
∆LNFD	−5.5814 (0.0000)	−6.4409 (0.0000)	58.721 (0.0000)	72.393 (0.0000)
∆LNUP	−1.7899 (0.0367)	−2.9082 (0.0018)	24.616 (0.0061)	51.969 (0.0000)
∆LNEC	−9.6853 (0.0000)	−8.5148 (0.0000)	75.763 (0.0000)	87.362 (0.0000)
∆LNDS	−7.4301 (0.0000)	−7.4811 (0.0000)	65.838 (0.0000)	58.383 (0.0000)
∆LNLF	−1.3869 (0.0827)	−3.3971 (0.0003)	38.309 (0.0000)	37.881 (0.0000)
∆LNINF	−4.0941 (0.0000)	−3.9802 (0.0000)	33.514 (0.0002)	32.70 (0.0003)
∆LNEX	−8.0543 (0.0000)	−7.545 (0.0000	66.547 (0.0000)	81.973 (0.0000)
∆LNTD	−8.2230 (0.0000)	−7.2176 (0.0000)	62.655 (0.0000)	62.348 (0.0000)

**Table 4:** Results for Panel of SAARC Countries.

Variables	Model-3	Model-4	Model-5
	LNCO_2_	LNGDPP	LNFDI
LNCO_2_	-	0.0977 (0.5549)	0.2557 (0.7432)
LNGDPP	0.3188 *** (0.0615)	-	0.6104 (0.4153)
LNFDI	0.0547 (0.2404)	−0.0187 (0.4348)	-
LNFD	0.1234 (0.6788)	-	-
LNUP	−0.2979 ** (0.0461)	-	-
LNEC	−0.6512 * (0.0014)	-	-
LNDS	-	1.3163 * (0.000)	10.115 * (0.0006)
LNLF	-	−0.0572 (0.3864)	0.3912 (0.5051)
LNINF	-	0.9188 * (0.000)	-
LNEX	-	-	−3.5413 (0.1343)
LNTD	-	-	−1.3357 (0.2743)
Adj R-Squared	0.8933	0.9681	0.9262

Note: *, **, and *** symbolize the 1%, 5%, and 10% levels respectively. Parenthesis consists of *p*-values.

**Table 5:** Results for Individual Countries. Bangladesh and India.

Variables	Bangladesh			India		
	Model-3	Model-4	Model-5	Model-3	Model-4	Model-5
	LNCO_2_	LNGDPP	LNFDI	LNCO_2_	LNGDPP	LNFDI
LNCO_2_	-	−1.2366 (0.3669)	−4.3554 ** (0.0354)	-	2.6166 * (0.0000)	5.7379 ** (0.0410)
LNGDPP	0.7196 * (0.0006)	-	5.0376 * (0.0033)	0.7386 ** (0.0113)	-	−0.3986 (0.8274)
LNFDI	−0.0243 ** (0.0201)	−0.0356 (0.8184)	-	−0.0571 *** (0.0727)	−0.0376 (0.2514)	-
LNFD	1.5958 * (0.0000)	-	-	−0.2733 (0.1783)	-	-
LNUP	0.5636 * (0.0029)	-	-	−0.6198 (0.1463)	-	-
LNEC	−2.2198 * (0.0000)	-	-	−0.3086 *** (0.0842)	-	-
LNDS	-	0.50157 (0.8362)	12.857 * (0.0001)	-	1.0354 * (0.0001)	3.4733 (0.2997)
LNLF	-	−0.3435 (0.5509)	−2.0228** (0.0326)	-	0.2692 * (0.0000)	1.4559 * (0.0047)
LNINF	-	2.2886 *** (0.0643)	-	-	−0.4122 *** (0.0688)	-
LNEX	-	-	−4.2971 ** (0.0159)	-	-	−3.6156 (0.2405)
LNTD	-	-	−0.7095 (0.5577)	-	-	0.15103 (0.9425)
Adj R2	0.9979	0.9892	0.9318	0.9928	0.9766	0.9086

Note: *, **, and *** symbolize the 1%, 5%, and 10% levels respectively. Parenthesis consists of *p*-values.

**Table 6:** Results for the Individual Countries. Nepal, Pakistan, and Sri Lanka.

Variables	Nepal			Pakistan			Sri Lanka		
	Model-3	Model-4	Model-5	Model-3	Model-4	Model-5	Model-3	Model-4	Model-5
	LNCO_2_	LNGDPP	LNFDI	LNCO_2_	LNGDPP	LNFDI	LNCO_2_	LNGDPP	LNFDI
LNCO_2_	-	0.3250 (0.4347)	2.8042 (0.5915)	-	0.5897 (0.4419)	10.266 * (0.0000)	-	−0.6852 (0.1021)	−0.3222 (0.6007)
LNGDPP	0.4158 (0.1851)	-	−2.9404 (0.6390)	0.1317 (0.1731)	-	−1.3627 ** (0.0156)	0.8364 * (0.0009)	-	1.4789 *** (0.0522)
LNFDI	0.0105 (0.4547)	−0.0138 (0.5312)	-	0.0397 (0.1412)	−0.0319 (0.6506)	-	−0.5519 ** (0.0221)	−0.5092 (0.1516)	-
LNFD	0.4194 ** (0.0492)	-	-	0.08 (0.4744)	-	-	0.5413 ** (0.0162)	-	-
LNUP	0.0128 (0.9460)	-	-	−0.6035 * (0.0011)	-	-	0.0920 (0.2002)	-	-
LNEC	−1.0531 * (0.0091)	-	-	−0.2408 *** (0.0553)	-	-	0.4312 (0.3776)	-	-
LNDS	-	−1.4334 (0.1543)	−3.0811 (0.8069)	-	0.8063 *** (0.0562)	3.112 * (0.0000)	-	2.2608 ** (0.0390)	−0.4477 (0.6728)
LNLF	-	0.3451 (0.1994)	−1.156 (0.8426)	-	0.1537 (0.1820)	0.7336 * (0.0040)	-	0.1629 (0.3634)	−0.6037 (0.3111)
LNINF	-	1.1028 *** (0.0596)	-	-	0.6021 * (0.0030)	-	-	1.7707 * (0.0086)	-
LNEX	-	-	13.166 (0.3804)	-	-	1.3659 *** (0.0573)	-	-	2.1845 (0.1957)
LNTD	-	-	1.8232 (0.8768)	-	-	1.1612 ** (0.0267)	-	-	2.2973 ** (0.0449)
Adj R2	0.9580	0.9734	0.0721	0.9463	0.9637	0.8924	0.9515	0.9953	0.8407

Note: *, **, and *** symbolize the 1%, 5%, and 10% levels respectively. Parenthesis consists of *p*-values.

**Table 7:** Results of Panel Co-Integration Tests (Engle-Granger Based).

Methods		Model−3	Model-4	Model-5
		LNCO_2_	LNEG	LNFDI
**Pedroni**				
Within dimension	Panel v-Stat	0.0119 (0.4952)	−0.7505 (0.7735)	−2.3785 (0.9913)
	Panel rho-Stat	0.1422 (0.5565)	1.1109 (0.8667)	0.3014 (0.6185)
	Panel PP-Stat	−2.6846 * (0.0036)	−0.4312 (0.3332)	−14.162 * (0.0000)
	Panel ADF-Stat	−2.6241* (0.0043)	−1.5672 ** (0.0585)	−7.6142 * (0.0000)
Between dimension	Group rho-Stat	0.7305 (0.7675)	1.8676 (0.9691)	1.5663 (0.9414)
	Group PP-Stat	−5.6019 * (0.000)	−0.4316 (0.3330)	−7.8117 * (0.0000)
	Group ADF-Stat	−4.8649 * (0.000)	−2.7071 * (0.0034)	−5.4417 * (0.0000)
Kao	ADF t-Stat	−3.1415 * (0.0008)	−3.1835 * (0.0007)	−3.6693 * (0.0001)

Note: *, **, and 10% levels respectively. Parenthesis consists of *p*-values.

**Table 8:** Results of Panel Vector Error Correction Model (PVECM) for the Model (3).

Variables	Model-6	Model-7	Model-8	Model-9	Model-10	Model-11
	ΔLNCO_2_	ΔLNGDPP	ΔLNFDI	ΔLNFD	ΔLNUP	ΔLNEC
ECT	0.003 (0.6730)	0.0119 ** (0.0721)	0.1367 (0.5152)	−0.0352 * (0.0047)	0.1035 * (0.0006)	0.0093 * (0.0005)
∆LNCO_2_	-	0.0976 (0.2629)	−3.1179 (0.2651)	−0.0216 (0.89410	0.2349 (0.5481)	0.0177 (0.6065)
∆LNGDPP	0.0056 (0.9553)	-	2.343 (0.4301)	0.34675 ** (0.0463)	−0.5718 (0.1705)	−0.0241 (0.5098)
∆LNFDI	0.01406 * (0.0001)	−0.0012 (0.7034)	-	0.0007 (0.9079)	−0.0007 (0.9632)	−0.00009 (0.9423)
∆LNFD	0.1804 * (0.0015)	0.0083 (0.8711)	1.4537 (0.3771)	-	−0.4468 *** (0.0542)	0.05996 * (0.0037)
∆LNUP	0.0624 * (0.0065)	−0.0204 (0.3259)	−0.2351 (0.7241)	0.0172 (0.6566)	-	0.0326 * (0.0001)
∆LNEC	0.318 (0.2858)	−0.1783 (0.5146)	−5.9129 (0.5006)	−0.1377 (0.7872)	−2.7453 ** (0.0272)	-
F-test	5.5245	1.0981	3.9109	1.3684	4.4885	3.8428

Note: *, **, and *** symbolize the 1%, 5%, and 10% levels respectively. Parenthesis consists of *p*-values.

**Table 9:** Variance Decomposition of LNCO2.

Period	LNCO_2_	LNGDPP	LNFDI	LNFD	LNUP	LNEC
1	100	0.00	0.00	0.00	0.00	0.00
2	85.232	0.095	5.855	6.278	2.085	0.455
3	84.146	0.496	4.178	6.927	1.934	2.319
4	81.791	0.609	4.908	8.626	2.172	1.895
5	81.308	0.839	4.578	9.101	2.217	1.957
6	80.352	0.946	4.788	9.721	2.404	1.789
7	80.333	0.997	4.670	9.662	2.470	1.868
8	80.000	1.028	4.877	9.707	2.592	1.795
9	80.071	1.044	4.894	9.499	2.661	1.831
10	79.985	1.049	5.051	9.358	2.756	1.802

## Data Availability

Data from World Development Indicators (WDI), SAARC energy outlook 2030, the international monetary fund (IMF), and Bruegel Dataset.
